# ADHERENCE TO INTERVENTIONS AT A FALL CLINIC: PRELIMINARY DESCRIPTIVE DATA FROM A SINGLE ACADEMIC INSTITUTION

**DOI:** 10.1093/geroni/igad104.2272

**Published:** 2023-12-21

**Authors:** Yuka Shichijo, Fred Ko, Renee Slon, Nisha Rughwani, Yuji Yamada

**Affiliations:** Mount Sinai Beth Israel, New York City, New York, United States; Icahn School of Medicine at Mount Sinai, New York City, New York, United States; Mount Sinai Morningside, New York City, New York, United States; Icahn School of Medicine at Mount Sinai, New York City, New York, United States; Icahn School of Medicine at Mount Sinai, New York City, New York, United States

## Abstract

**Background:**

Falls and fall-related injuries are major public health concerns. Approximately 50 billion dollars are spent on annual medical costs related to falls. Fall prevention is a priority for promoting active and healthy aging in older adults. Fall prevention clinics enable focused evaluation, identification, and treatment of modifiable risk factors, specifically for falls; however, their effectiveness is dependent upon patient adherence to preventive interventions.

**Methods:**

We analyzed patients aged > 65 years who visited our fall clinic between February 2021 and February 2023. We took an interdisciplinary approach to improve adherence to recommendations, including follow-up phone calls by a dedicated nurse to confirm adherence, nurse-directed interventions such as Tai Chi sessions, and home environment assessments by a social worker.

**Results:**

48 patients were included in this analysis, with a mean age of 84 (65-99), and 85% were female. Fall-specific risk factors identified included impairment of strength, gait, or balance (96%); use of fall-risk-increasing medications (54%); problems with feet affecting gait (40%); visual impairment (29%); and postural hypotension (6%). The adherence rates for our interventions were 48% for physical therapy referrals, 28% for podiatry referrals, 36% for ophthalmology referrals, 47% for other referrals, 75% for vitamin D prescriptions, and 50% for assistive device prescriptions.

**Conclusions:**

There is room for improvement in adherence to referrals. A larger sample size and longer follow-up period are required to investigate factors related to non-adherence. The delivery of both verbal and written recommendations, the identification of each patient’s barriers, and same-day referrals may help improve adherence.

